# Severe community-acquired *Streptococcus pneumoniae* bacterial meningitis: clinical and prognostic picture from the intensive care unit

**DOI:** 10.1186/s13054-023-04347-3

**Published:** 2023-02-23

**Authors:** María Martín-Cerezuela, Maialen Aseginolaza-Lizarazu, Patricia Boronat-García, María José Asensio-Martín, Gisela Alamán-Laguarda, Francisco Álvarez-Lerma, David Roa-Alonso, Lorenzo Socias, Paula Vera-Artázcoz, Paula Ramírez-Galleymore, the Grupo de Trabajo en Infección y Sepsis (GTEIS) from the Sociedad Española de Medicina Intensiva y Unidades Coronarias (SEMICYUC), Bárbara Balandin-Moreno, Loreto Vidaur-Tello, Silvia Sánchez-Morcillo, Juan Carlos Ballesteros-Herráez, Sergio Ossa-Echeverri, David Andaluz-Ojeda, Miguel Ángel Blasco-Navalpotro, Ana Abella-Álvarez, Leonor Nogales-Martín, Emili Díaz-Santos, Oriol Plans-Galván, Isabel Conejo-Márquez

**Affiliations:** 1 Intensive Care Unit, Instituto de Investigación Sanitaria La Fe, Hospital Universitario y Politécnico La Fe, Valencia, Spain; 2 Intensive Care Unit, Hospital Donostia-Donostia Ospitalea, Guipúzcoa, Spain; 3 Intensive Care Unit, Hospital Univeristari Germans Trias i Pujol, Badalona, Spain; 4 Intensive Care Unit, Hospital Universitario La Paz, Madrid, Spain; 5 Intensive Care Unit, Hospital Universitario de la Ribera, Valencia, Spain; 6 Intensive Care Unit, Fundación, Instituto Hospital del Mar de Investigaciones Médicas (IMIM), Barcelona, Spain; 7 Intensive Care Unit, Hospital Severo Ochoa, Madrid, Spain; 8 Intensive Care Unit, Hospital Son Llàtzer, Mallorca, Spain; 9 Intensive Care Unit, Institut d’Investigació Biomèdica Sant Pau (IIB SANT PAU), Hospital de la Santa Creu i Sant Pau, Barcelona, Spain; 10 Intensive Care Unit, Hospital Universitario y Politécnico La Fe, Avenida Fernando Abril Martorell, nº 106, CP 46026 Valencia, Spain

**Keywords:** Meningitis, *Streptococcus pneumoniae*, Intensive care units, Critically illness

## Abstract

**Background:**

Severe community-acquired pneumococcal meningitis is a medical emergency. The aim of the present investigation was to evaluate the epidemiology, management and outcomes of this condition.

**Methods:**

This was a retrospective, observational and multicenter cohort study. Sixteen Spanish intensive care units (ICUs) were included. Demographic, clinical and microbiological variables from patients with *Streptococcus pneumoniae* meningitis admitted to ICU were evaluated. Clinical response was evaluated at 72 h after antibiotic treatment initiation, and meningitis complications, length of stay and 30-day mortality were also recorded.

**Results:**

In total, 255 patients were included. Cerebrospinal fluid (CSF) culture was positive in 89.7%; 25.7% were non-susceptible to penicillin, and 5.2% were non-susceptible to ceftriaxone or cefotaxime. The most frequent empiric antibiotic regimen was third-generation cephalosporin (47.5%) plus vancomycin (27.8%) or linezolid (12.9%). A steroid treatment regimen was administered to 88.6% of the patients. Clinical response was achieved in 65.8% of patients after 72 h of antibiotic treatment. Multivariate analysis identified two factors associated with early treatment failure: invasive mechanical ventilation (OR 10.74; 95% CI 3.04–37.95, *p* < 0.001) and septic shock (OR 1.18; 95% CI 1.03–1.36, *p* = 0.017). The 30-day mortality rate was 13.7%. Only three factors were independently associated with 30-day mortality: delay in start of antibiotic treatment (OR 18.69; 95% CI 2.13–163.97, *p* = 0.008), Sepsis-related Organ Failure Assessment (SOFA) score (OR 1.36; 95% CI 1.12–1.66, *p* = 0.002) and early treatment failure (OR 21.75 (3.40–139.18), *p* = 0.001). Neurological complications appeared in 124 patients (48.63%).

**Conclusions:**

Mortality rate in critically ill patients with pneumococcal meningitis is lower than previously reported. Delay in antibiotic treatment following admission is the only amendable factor associated with mortality.

## Introduction

Community-acquired acute bacterial meningitis (CABM) remains one of the most feared infectious diseases due to its high morbidity and mortality, especially when its severity requires intensive care unit (ICU) admission. *Streptococcus pneumoniae* is the most frequent causative agent of CABM in adults, responsible for 75–80% of all bacterial meningitis cases [1]. Despite ongoing advances in diagnostic, treatment and vaccine strategies, mortality remains up to 15% in all *S. pneumoniae* CABM cases and rises to 33% in the critically ill population [2–5]. Prognostic factors comprise early recognition, degree of severity at admission and appropriate and immediate antibiotic administration [3].

Classically, a combination of β-lactam (mainly a third-generation cephalosporin) and vancomycin (in case of high local rates of resistance to penicillin) is the standard of antimicrobial care [6–8]. Some good results have been obtained with newer antibiotics such as linezolid and ceftaroline, but its use is not endorsed by any strong scientific evidence [9, 10]. Adjunctive treatment with dexamethasone is the second key component in *S. pneumoniae* CABM treatment, as it improves both mortality and neurological outcome [11, 12]. However, its use and effect have not been described in recent publications including CABM patients admitted to ICU.

Despite the clinical and social impact of CABM caused by *S pneumoniae*, there is a deficit of current publications on routine medical practices and the prognosis of patients requiring ICU admission [2, 3, 13–17]. Therefore, we designed this retrospective, multicenter study with the aim to examine current epidemiology and management of the disease in Spanish ICUs.

## Methods

### Data collection and review procedure

This retrospective, observational and multicenter study was endorsed by the Infection and Sepsis Working Group of the Spanish Society of Intensive Medicine. All *S. pneumoniae* meningitis episodes diagnosed from January 2010 to December 2018 at 16 Spanish ICUs were included. Demographic, clinical and microbiological variables were extracted from electronic medical records systems. The Hospital Ethics Committee reviewed and approved the project at each of the participating hospitals, as well as the Spanish Medicines Agency; informed consent was waived due to the retrospective nature of the study.

Meningitis was defined by the presence of a compatible clinical picture (headache, fever, stiff neck and/or alteration of mental status) and microbiologic confirmation reached by means of *S. pneumoniae* isolation in CSF, or *S. pneumoniae* antigen or DNA detection in CSF [6, 8]. All *S. pneumoniae* isolates were identified by standard laboratory methods in each hospital’s clinical microbiology laboratory. Antimicrobial susceptibility of isolates was determined by disk diffusion test, automated systems, E-tests or microdilution tests. *S. pneumoniae* was considered susceptible to penicillin if the minimum inhibitory concentration (MIC) was 0.06 μg/mL or less, and resistant if the MIC was more than 0.06 μg/mL. Isolates were considered susceptible to third-generation cephalosporin if the cefotaxime or ceftriaxone MIC was ≤ 0.5 μg/mL, in accordance with the criteria of European Committee on Antimicrobial Susceptibility Testing [19].

All treatment variables were collected. Empirical treatment was that which was initiated upon clinical suspicion and before microbiological results are available. It was considered de-escalated when, based on the microbiological results, the spectrum or number of antimicrobials was reduced. Also, dosages and duration of the antibiotic treatments were registered. A delay in antibiotic treatment was considered to have occurred if the first dose was administered 6 h or more after hospital admission [20]. Other cutoff points were also assessed. Antibiotic treatment was considered to be appropriate when causative pathogens were susceptible to at least one prescribed antibiotic. Adjuvant therapy with steroids was also recorded. A delay in steroid treatment was considered to have occurred if treatment was not started before or at the same time than antibiotics.

Clinical response was evaluated at 72 h after antibiotic treatment initiation. Patients were considered to be responders if clinical improvement was observed, and all signs and symptoms present at the time of diagnosis had improved or disappeared. Treatment failure was defined as persistence or worsening of the signs and symptoms of infection despite antimicrobial treatment, or death of the patient directly related to the meningitis episode. Microbiologic response and CSF inflammatory parameters were evaluated in those patients for whom a second CSF sample was obtained.

Meningitis complications were identified according to the European Society of Clinical Microbiology and Infectious Diseases guidelines for bacterial meningitis and collected from medical records [8]. ICU and hospital length of stay and 30-day mortality were also recorded.

### Statistical analysis

Categorical variables were summarized as absolute numbers and percentages and analyzed with chi-square or Fisher’s exact test when indicated. Continuous variables were reported as mean and standard deviation or median and interquartile range (IQR), depending on their homogeneity, and were compared using Student’s t test or the Mann–Whitney U test as appropriate. For all comparisons, values of *p* ≤ 0.05 were considered statistically significant. A multivariate logistic regression model adjusted by Bonferroni correction was developed to identify the variables associated with patient mortality and early treatment failure. The statistical analysis was performed using the STATA® v.14.2 software.

## Results

### Clinical and demographic characteristics

A total of 255 critically ill patients with *S. pneumoniae* CABM from 16 centers were included in the study. The demographic and clinical characteristics of the included patients are shown in Table 1. Cerebral computer tomography was performed in 249 patients (97.65%).

**Table 1:** Characteristics of the study population

Variables	Survivors (*n* = 220)	Non-survivors (*n* = 35)	*p* value
Age (years) (mean ± SD)	60.21 ± 0.96	61.91 ± 2.96	0.527
Male gender (*n*, %)	134 (60.91)	23 (65.71)	0.587
*Comorbidities (n, %)*			
Arterial hypertension	102 (46.36)	13 (37.14)	0.309
Diabetes mellitus	54 (24.55)	8 (22.86)	0.829
Chronic heart disease	30 (13.64)	5 (14.29)	0.917
Chronic renal failure	11 (5)	3 (8.57)	0.389
Immunosuppression	27 (12.27)	3 (0.09)	0.528
HIV	6 (2.73)	3 (8.57)	0.082
*Predisposing conditions (n, %)*			
Otitis	85 (38.64)	7 (20.00)	0.033
Sinusitis	29 (13.18)	4 (11.43)	0.774
Vaccine against pneumococcus	7 (3.18)	1 (2.86)	0.918
Hospital admission in previous month	21 (9.55)	3 (8.57)	0.855
Prior neurological symptomatology	46 (19.09)	2 (5.71)	0.033
Clinical data at diagnosis			
Glasgow coma score (median, IQR)	10 (8–12)	8 (4–9)	< 0.001
Glasgow < 8 (indicating coma) (*n*, %)	80 (36.36)	22 (62.86)	0.003
APACHE-II score (median, IQR)	16 (12–21)	23 (18–28)	< 0.001
SOFA score (median, IQR)	4 (3–6)	7 (5–11)	< 0.001
SAPS3 score (median, IQR)	55.5 (46–63)	64 (48–75)	0.002
Septic shock (*n*, %)	28 (12.73)	11 (31.43)	0.004
Mechanical ventilation (*n*, %)	138 (62.73)	31 (88.57)	0.003
Length of ICU stay (days) (median, IQR)	7 (4–13)	5 (2–13)	0.081
Length of hospital stay (days) (median, IQR)	16 (13–31)	6 (2–13)	< 0.001

### Microbiological and biochemical results

Characteristics of microbiological isolates and biochemical results are shown in Table 2. Non-survivors had lower CSF glucose and higher levels of CSF proteins; their serum procalcitonin was higher, and lymphocyte count was lower in their blood samples.

**Table 2:** Biochemical and microbiological data

Variables	Survivors (*n* = 220)	Non-survivors (*n* = 35)	*p* value
*CSF data*			
Glucose (mg/dl),median (IQR)	5 (0.4–39)	1.5 (0–10)	0.019
White cell counts (/μL), median (IQR)	2008 (478–6115)	1360 (225–4800)	0.254
Polymorphonuclears (/μL), median (IQR)	1972 (472–5978)	1068 (152–4560)	0.105
Protein (mg/dl), median (IQR)	451 (280–680)	621 (382–1116)	0.003
Culture CSF, *n* positive/*n* evaluated (%)	194/218 (88.99)	33/35 (94.29)	0.338
*Streptococcus pneumoniae* penicillin-non-susceptible, *n*/*n* evaluated (%)	41/154 (26.62)	5/25 (20.00)	0.482
*Streptococcus pneumoniae* ceftriaxone-non-susceptible, *n*/*n* evaluated (%)	7/106 (0.06%)	0/20 (0%)	0.253
*Streptococcus pneumoniae* linezolid-non-susceptible, *n*/*n* evaluated (%)	0/44 (0%)	0/10 (0%)	*
*Streptococcus pneumoniae* vancomycin-non-susceptible, *n*/*n* evaluated (%)	0/86 (0%)	0/12 (0%)	*
*Streptococcus pneumoniae*-DNA, *n* positive/*n* evaluated (%)	28/71 (39.44)	5/13 (38.46)	0.947
Soluble *Streptococcus pneumoniae*-antigen, *n* positive/*n* evaluated (%)	80/116 (68.97)	11/18 (61.11)	0.507
*Serum data*			
White cell counts (× 10^3^/μL) median (IQR)	17,620 (12,595–23,450)	14,000 (8000–25,600)	0.308
Polymorphonuclears(× 10^3^/μL), median (IQR)	16,110 (10,900–21,540)	12,724 (7680–24,000)	0.305
Lymphocytes (× 10^3^/μL), median (IQR)	690 (400–1060)	540 (290–714)	0.048
C-reactive protein (mg/L), median (IQR)	86 (27–227)	161 (28–258)	0.268
Procalcitonin (ng/mL), median (IQR)	6.6 (1.7–16.0)	11.0 (4.6–23)	0.034
Blood culture, *n* positive/*n* evaluated (%)	131/193 (67.88)	18/29 (62.07)	0.535

The * means that it cannot be calculated because no patients were detected

CSF culture was positive in 227 patients (89.7%); 25.7% (*n* = 46) were non-susceptible to penicillin, and 5.2% (*n* = 7) were non-susceptible to ceftriaxone/cefotaxime. No strain was resistant to linezolid or vancomycin. *S. pneumoniae*-DNA was positive in 39.3% of CSF samples, and soluble *S. pneumoniae*-antigen was positive in 67.9% of CSF samples. Blood cultures were performed in 222 patients (87.0%) and were positive for *S. pneumoniae* in 149 cases (67.1%).

### Treatment

Treatment characteristics are reported in Table 3. The most frequent initial antibiotic regimens consisted of third-generation cephalosporin alone (47.45%) for a median of 11 (7–14) days, plus vancomycin (27.84%) for a median of 9 (3.0–13.5) days or plus linezolid (12.94%) for a median of 11 (8–15) days. Other regimens were observed in 30 patients (11.7%) and included carbapenems (20 patients), beta-lactam/beta-lactamase inhibitor (6 patients) or fluoroquinolones (4 patients). Treatment was combined with ampicillin (28.24%) or acyclovir (5.10%); 11.76% received other combinations of antibiotics. All patients received appropriate initial empiric therapy, except for five patients who received ceftriaxone for non-susceptible *S. pneumonia,* all of whom survived*.*

**Table 3:** Treatment and neurological complications

Variables	Survivors (*n* = 220)	Non-survivors (*n* = 35)	*p* value
*Treatment*			
Corticosteroids (*n*, %)	197 (89.50)	29 (82.86)	0.254
Third-generation cephalosporin alone (*n*, %)	101 (45.91)	20 (57.14)	0.274
Plus vancomycin regimen (*n*, %)	62 (28.18)	9 (25.71)	0.841
Plus linezolid regimen (*n*, %)	28 (12.73)	5 (14.29)	0.788
Other regimens (*n*, %)	29 (13.18)	1 (2.86)	0.092
Delay 2 h in starting antibiotic therapy (*n*, %)	64 (29.09)	13 (37.14)	0.105
Delay 6 h in starting antibiotic therapy (*n*, %)	6 (2.73)	4 (11.43)	0.023
Delay in starting steroid treatment (*n*, %)	19 (8.64)	8 (22.86)	0.011
*Neurological complications*	92 (41.82)	32 (91.42)	<0.001
Brain abscess (*n*, %)	7 (3.18)	2 (5.71)	0.435
Cerebritis (*n*, %)	12 (5.45)	3 (8.57)	0.442
Epilepsy (*n*, %)	36 (16.36)	9 (25.71)	0.151
Ischemic stroke (*n*, %)	24 (10.91)	6 (17.14)	0.228
Brain edema (*n*, %)	23 (10.45)	14 (40.00)	< 0.001

Systemic corticosteroids were administered in 88.63% of patients for a median of 4 (3–7) days. Steroids were administered from the day of admission in 89.4% of cases. In 27 patients, steroid administration was delayed (initiated after antibiotic treatment when pneumococcal etiology was confirmed). Steroid dosage agreed in all cases with the European Society of Clinical Microbiology and Infectious Diseases recommendations: 10 mg every 6 h for 4 days [8].

### Outcome

After 72 h of starting of antibiotic treatment, clinical improvement was observed in 65.88% of the patients. Antibiotic therapy was modified at 72 h in 52 patients (20.39%): de-escalation in 75% and escalation in 25% (due to lack of clinical improvement).

Systemic complications were renal failure that required continuous renal replacement therapy in 8 patients (3.14%) and septic shock in 39 patients (15.29%). Mechanical ventilation was needed in 169 patients (66.27%) due to low GCS punctuation (Table 1).

Neurological complications appeared in 124 patients (48.63%); the types of complications and their distribution among survivors and non-survivors are reported in Table 3. Sinus vein thrombosis, subdural empyema or hemorrhagic infarction were not identified in medical charts.

Factors associated with early clinical response and 30-day mortality are reported in Table 4. Early treatment failure occurred in 87 patients (34.1%). Age and severity were identified as risk factors for treatment failure in the univariate analysis (Table 4), but only invasive mechanical ventilation (OR 10.74; 95% CI 3.04–37.95, *p* < 0.001) and septic shock (OR 1.18; 95% CI 1.03–1.36, *p* = 0.017) persisted as a risk factor in the multivariate analysis.

**Table 4:** Factors independently associated with clinical improvement at 72 h and 30-day mortality in univariate analysis of patients with *Streptococcus pneumoniae* meningitis

Factors	Failure of treatment at 72 h		30-day mortality	
	OR (95% CI)	*p* value	OR (95% CI)	*p* value
Age	1.02 (1.00–1.04)	0.020	1.01 (0.98–1.03)	0.525
Male	1.20 (0.70–2.05)	0.509	1.23 (0.58–2.60)	0.588
*Predisposing conditions*				
Otitis	0.77 (0.45–1.33)	0.352	0.40 (0.17–0.95)	0.038
Sinusitis	1.12 (0.522–2.40)	0.771	0.85 (0.28–2.58)	0.774
HIV	0.54 (0.11–2.66)	0.450	3.35 (0.80–14.04)	0.099
Prior neurological symptomatology	0.76 (0.38–1.50)	0.423	0.23 (0.05–0.99)	0.049
Glasgow score	0.77 (0.70–0.85)	< 0.001	0.76 (0.67–0.86)	< 0.001
Glasgow < 8	3.25 (1.90–5.57)	< 0.001	2.96 (1.41–6.20)	0.004
APACHE-II score	1.14 (1.09–1.19)	< 0.001	1.14 (1.08–1.20)	< 0.001
SOFA score	1.44 (1.28–1.63)	< 0.001	1.42 (1.25–1.63)	< 0.001
SAPS3 score	1.09 (1.05–1.12)	< 0.001	1.05 (1.01–1.08)	0.006
Septic shock	4.44 (2.16–9.09)	< 0.001	3.14 (1.39–7.11)	0.006
Mechanical ventilation	19.78 (6.94–56.42)	< 0.001	4.61 (1.57–13.51)	0.005
*CSF data*				
Glucose	1.00 (0.99–1.00)	0.140	0.98 (0.97–1.00)	0.046
White cell counts	1.00 (1.00–1.00)	0.921	1.00 (1.00–1.00)	0.635
Polymorphonuclears	1.00 (1.00–1.00)	0.950	1.00 (1.00–1.00)	0.831
Protein	1.00 (1.00–1.00)	< 0.001	1.00 (1.00–1.00)	0.001
*Serum data*				
White cell counts	1.00 (1.00–1.00)	0.781	1.00 (1.00–1.00)	0.366
Polymorphonuclears	1.00 (1.00–1.00)	0.465	1.00 (1.00–1.00)	0.587
Lymphocytes	1.00 (1.00–1.00)	0.624	1.00 (1.00–1.00)	0.217
C-reactive protein	1.00 (1.00–1.00)	0.625	1.00 (1.00–1.00)	0.785
Procalcitonin	1.01 (1.00–1.03)	0.080	1.01 (1.00–1.02)	0.097
Third-generation cephalosporin alone	0.69 (0.41–1.16)	0.163	1.57 (0.76–3.23)	0.219
Plus vancomycin regimen	1.38 (0.78–2.43)	0.267	0.88 (0.39–1.99)	0.762
Plus linezolid regimen	1.50 (0.71–3.17)	0.283	1.14 (0.41–3.19)	0.799
Other regimens	0.81 (0.35–1.85)	0.613	0.19 (0.26–1.47)	0.112
Delay start antibiotic therapy	0.81 (0.22–2.96)	0.746	5.43 (1.41–20.91)	0.014
Corticosteroids	1.41 (0.60–3.33)	0.432	0.56 (0.21–1.50)	0.252
Delay in starting steroids treatment	1.87 (0.83–4.21)	0.130	3.57 (1.39–9.15)	0.008
Early failure treatment	-	-	10.92 (4.53–26.32)	< 0.001

Thirty-five patients had died at the study endpoint (13.73%). There were no differences concerning clinical or demographic characteristics between survivors and non-survivors. In 114 patients, the antibiotic was administered in the first two hours, but this fact was not accompanied by significant differences in mortality (9.6% vs. 16%; *p* = 0.105). In 183 patients, the antibiotic was administered in the first six hours. For this cutoff point, statistically significant differences were found (10.9% vs 40%; *p* = 0.023). As expected, severity scores (Acute Physiology and Chronic Health disease Classification System II (APACHE-II), Sepsis-related Organ Failure Assessment (SOFA) and Simplified Acute Physiology Score III (SAPS3)) were significantly higher in patients who died. GLASGOW score and the percentage of patients in septic shock or under mechanical ventilation also differed between the two groups. In the multivariate analysis, delay in antibiotic treatment (OR 18.69; 95% CI 2.13–163.97, *p* = 0.008), SOFA score (OR 1.36; 95% CI 1.12–1.66, *p* = 0.002) and early treatment failure (OR 21.75 (3.40–139.18), *p* = 0.001) were independently associated with mortality.

Clinical follow-up after hospital discharge detected five patients with hearing loss and one patient with third cranial nerve palsy as neurological sequelae.

## Discussion

Our study describes a large series of meningitis cases caused by *S. pneumoniae* in a Spanish population requiring ICU admission over a period of 8 years. Despite the fact that *S. pneumoniae* CABM carries terrible morbidity and mortality, publications concerning the critically ill patient are scarce and are mostly from the pre-steroid era [1–3, 13–17]. Remarkably, the mortality in our series is clearly lower than previously published.

Despite widespread use of vaccination in high- and medium-income countries, *S. pneumoniae* remains the most frequent etiology in CABM. Antibiotic susceptibility issues for *S. pneumoniae* are becoming more frequent, and some authors have related this fact to poorer prognosis [2]. However, the described relationship was not due to inappropriate empiric antibiotic treatment. Higher virulence or slower eradication of bacteria was other arguments put forward by the authors [3, 13]. In our series, 25% of strains were penicillin-resistant and 5% were ceftriaxone-resistant. Nevertheless, we did not find any prognostic relationship and appropriate antibiotic treatment was present in 98% of the cases.

*S. pneumoniae* CABM ICU mortality has been reported to range between 13 and 37%, with more recent data showing a mortality of around 17% [2, 3, 13–18]. Surprisingly, despite the fact that mortality is still excessively high, guidelines continue to recommend the same empirical antibiotic pattern that has been suggested since 2004 [6, 8]. Some potential advantages are attributed to relatively new drugs such as linezolid and ceftaroline. From the point of view of pharmacokinetics, linezolid would clearly achieve better concentrations in cerebrospinal fluid than vancomycin (ceftaroline would be at the same level as ceftriaxone) [21–23]. In animal models, ceftaroline has shown better bactericidal capacity and even a possible beneficial immunomodulatory effect due to inhibition of human cathelicidin IL-37 [24]. However, clinical studies are scarce, and only clinical series have shown potential benefits of the use of new antibiotics in CABM [9, 10]. In our study, 113 (44%) patients received vancomycin or linezolid. The use of either of these drugs, or any other antibiotic combination, was not associated with patient prognosis.

Epidemiological studies and clinical trials have identified steroid treatment recommendation as a landmark after which mortality decreased significantly [14]. In a recent publication, Koelman et al. reviewed the prognosis of 1783 patients with acute meningitis due to *S pneumoniae* collected in the Netherlands over a 20-year period. Dexamethasone was administered in 3% of patients in the period from 1998 to 2002 and in 82% of patients in the period from 2006 to 2018. Once again, the use of steroids was associated with lower mortality and lower frequency of complications and sequelae. However, the clinical benefit only occurs in those cases in which the steroid is administered before or in association with antibiotic treatment. In our series, steroid treatment was administered out of protocol in 27 patients (10.5%), a proportion similar to that published by Koelman et al. (8.1%) [17]. The reported reason for the delay was an inadvisable expectant attitude until the identification of the presence of *S pneumoniae.*

From our viewpoint, this is the first multicenter report of severe *S. pneumoniae* CABM ICU prognosis after widespread use of steroids. Severity, neurological involvement, empirical antibiotic strategy and rate of appropriateness, use of invasive mechanical ventilation and presence of septic shock have been similarly depicted in other studies including critically ill patients [2, 3, 12]. In our patients, steroid use, occurring in up to 88.6% of the cases in contrast to 19–55.5%, appears to be the main difference [3, 4, 18]. Of course, other improvements in patient management, such as respirator settings or therapeutic vasopressor algorithms, could also have influenced patient prognosis. Similarly, Buchholz et al*.* compared 54 patients admitted to their ICU between 2003 and 2015 to an historic cohort from their hospital (87 patients from 1984 to 2002); mortality dropped dramatically from 21.4 to 5.5% as steroid use increased from 18.4 to 85.5% [14].

Prognostic factors have previously been identified in *S. pneumoniae* CABM. In publications concerning critically ill patients, severity scores and a delay in antibiotic administration were independently associated with mortality [2–4, 16]. Our results agree with these findings and present antibiotic delay as the strongest predictor of death (OR 30.05; 95% CI 1.23–732.78, *p* = 0.037). Severity on admission is the main factor in death, which could be improved by early diagnosis of meningitis. Greater health literacy is needed for early identification of meningitis and consequently early initiation of treatment. Finally, as in other pathologies such as community-acquired pneumonia, early clinical response to treatment was associated with a better patient prognosis. In our study, only septic shock at presentation and need for mechanical ventilation were associated with early clinical response. Future studies could evaluate if other modifiable factors are associated with an early response and therefore could be implemented in the care of patients with CABM [25].

Our study has several limitations. The data were collected retrospectively; thus, factors that might have contributed to the patient prognosis could not be completely identified. Careful titration of sedation, keeping blood pressure in predefined corridors and prevention and detection of arising complications (especially those concerning nosocomial infections or neurological events) are examples of other clinically relevant issues. The impact of such interventions is extremely difficult to measure, and it is impossible to assess them in a retrospective study. Neurological complications were collected from medical charts, but no systematic pursuit was performed due to the retrospective nature of this study. Therefore, it is possible that some neurological complications have not been correctly identified in patients without control cerebral CT scans. Furthermore, we did not assess long-term outcome and neurological sequels.

In conclusion, *S. pneumoniae* CABM continues to be one of the most feared infectious diseases. However, critical care management and prognosis have not been deeply explored in the literature to date. In our large, multicenter series, we present an update on *S. pneumoniae* CABM prognosis. Fortunately, we have been able to corroborate an improvement in prognosis with respect to previous publications, as well as a possibility of better prognosis based on early initiation of antibiotic treatment.

## Data Availability

The datasets used in this study are available from the corresponding author on reasonable request.
